# Patient Safety in Surgery

**DOI:** 10.1186/s13037-014-0054-1

**Published:** 2015-02-26

**Authors:** James E Barone

**Affiliations:** Consultant, law firm of Silver, Golub, & Teitell, 184 Atlantic St., Stamford, CT 06901 USA

## ᅟ

As the pressure mounts on physicians to become more aware of patient safety, this timely new book appears (Box). The work is edited by Drs. Philip F. Stahel and Cyril Mauffrey, both of whom have published extensively on patient safety issues. Dr. Stahel is also the founding editor and Editor-in-Chief of the open-access journal *Patient Safety in Surgery*.

Experts in the field of patient safety from several countries contributed chapters. The list is diverse and includes surgeons, anesthesiologists, internists, nurses, liability insurance executives, risk managers, and importantly, patient advocates.

The book begins with a number of chapters devoted to such basics as measuring quality, describing and classifying different types of errors, communication, handoffs, accountability, and professionalism. Part two of the book deals with morbidity and mortality conferences, how to report and disclose complications, checklists, and insights into the performance of surgical quality improvement. Anesthesia, nursing, and patient family perspectives, and the challenges surrounding patient safety in the developing world make up the next section. The final chapters focus on case scenarios, one of which describes the preventable death of a young man after what tragically proved to be an unnecessary neurosurgical operation. Written by the parents of the patient, the many errors that occurred and the lack of transparency of the system are explored in detail.

The book is well organized with most chapters featuring an opening list of pitfalls and pearls, an outline of the problem, and specific “take-home” points. I particularly enjoyed the chapters on diagnostic errors, handovers, the surgical morbidity and mortality conference, and the management of unanticipated outcomes.

As is the case with most books having multiple contributors, some redundancies occur. For example, the same illustration of James Reason’s famous “Swiss Cheese” model of medical errors appears in two separate chapters. However, this does not take away from the importance of the work. The book can be read from cover to cover or used as a reference when specific questions about patient safety arise.

*Patient Safety in Surgery* is a valuable resource for patient care and quality improvement. I enthusiastically recommend this book to surgeons, nurses, and hospital administrators interested in improving the safety of surgical patients in their institutions. It would even be useful as a teaching tool for students and residents.

## Book information

*“Patient Safety in Surgery“.* Edited by Philip F. Stahel and Cyril Mauffrey; Springer, Heidelberg/New York; 1st edition, 2014. (ISBN: 978–1447143680)

